# 1-(2-Oxoindolin-3-yl­idene)-4-[2-(trifluoro­meth­oxy)phen­yl]thio­semi­carbazide

**DOI:** 10.1107/S1600536810034148

**Published:** 2010-08-28

**Authors:** Muhammad Ramzan, Humayun Pervez, M. Nawaz Tahir, Muhammad Yaqub, Mohammad S. Iqbal

**Affiliations:** a Department of Chemistry, Bahauddin Zakariya University, Multan 60800, Pakistan; b Department of Physics, University of Sargodha, Sargodha, Pakistan; c Department of Chemistry, Government College University, Lahore, Pakistan

## Abstract

The crystal structure of the title compound, C_16_H_11_F_3_N_4_O_2_S, is stabilized in the form of polymeric chains by N—H⋯O inter­actions. In the mol­ecular structure, two *S*(5) ring motifs are formed by intra­molecular N—H⋯N and N—H⋯O hydrogen bonding and two *S*(6) rings are present due to N—H⋯O and C—H⋯S inter­actions. π–π inter­actions are present with distances of 3.2735 (17), 3.563 (2) and 3.664 (4)/3.688 (3) Å between the centroids of the heterocyclic rings, between the centroids of the heterocyclic ring and trifluoro­meth­oxy-substituted phenyl ring, and between the centroids of the trifluoro­meth­oxy-substituted phenyl rings, respectively. The trifluoro­meth­oxy­phenyl group is disordered over two sites with an occupancy ratio of 0.642 (10):0.358 (10).

## Related literature

For our work on the synthesis of biologically important isatin (systematic name 1*H*-indole-2,3-dione) derivatives, see: Pervez *et al.* (2007 ▶, 2008 ▶, 2009 ▶). For a related structure, see: Pervez *et al.* (2010 ▶). For graph-set notation, see: Bernstein *et al.* (1995 ▶).
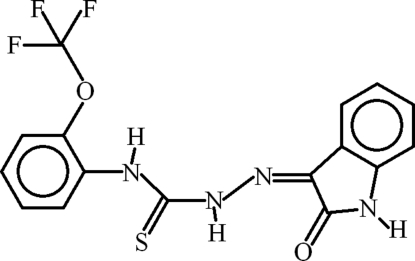


## Experimental

### 

#### Crystal data

C_16_H_11_F_3_N_4_O_2_S
*M* *_r_* = 380.35
Tetragonal, 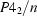

*a* = 13.4746 (8) Å
*c* = 18.1073 (10) Å
*V* = 3287.7 (3) Å^3^
*Z* = 8
Mo *K*α radiation
μ = 0.25 mm^−1^
*T* = 296 K
0.32 × 0.24 × 0.22 mm

#### Data collection

Bruker Kappa APEXII CCD diffractometer
Absorption correction: multi-scan (*SADABS*; Bruker, 2005 ▶) *T*_min_ = 0.937, *T*_max_ = 0.951
15673 measured reflections
4051 independent reflections
2321 reflections with *I* > 2σ(*I*)
*R*_int_ = 0.030

#### Refinement

*R*[*F* ^2^ > 2σ(*F* ^2^)] = 0.069
*wR*(*F* ^2^) = 0.202
*S* = 1.04
4051 reflections
242 parameters
46 restraints
H-atom parameters constrained
Δρ_max_ = 0.62 e Å^−3^
Δρ_min_ = −0.39 e Å^−3^

### 

Data collection: *APEX2* (Bruker, 2009 ▶); cell refinement: *SAINT* (Bruker, 2009 ▶); data reduction: *SAINT*; program(s) used to solve structure: *SHELXS97* (Sheldrick, 2008 ▶); program(s) used to refine structure: *SHELXL97* (Sheldrick, 2008 ▶); molecular graphics: *ORTEP-3 for Windows* (Farrugia, 1997 ▶) and *PLATON* (Spek, 2009 ▶); software used to prepare material for publication: *WinGX* (Farrugia, 1999 ▶) and *PLATON*.

## Supplementary Material

Crystal structure: contains datablocks global, I. DOI: 10.1107/S1600536810034148/bq2229sup1.cif

Structure factors: contains datablocks I. DOI: 10.1107/S1600536810034148/bq2229Isup2.hkl

Additional supplementary materials:  crystallographic information; 3D view; checkCIF report

## Figures and Tables

**Table 1:** Hydrogen-bond geometry (Å, °)

*D*—H⋯*A*	*D*—H	H⋯*A*	*D*⋯*A*	*D*—H⋯*A*
N1—H1⋯O1^i^	0.86	1.99	2.841 (3)	173
N3—H3⋯O1	0.86	2.07	2.748 (3)	136
N4—H4*A*⋯O2*A*	0.86	2.20	2.607 (8)	109
N4—H4*A*⋯N2	0.86	2.13	2.583 (4)	112
C11*A*—H11*A*⋯S1	0.93	2.40	3.096 (4)	132

Symmetry code: (i) 
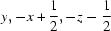
.

**Fig. 1.**
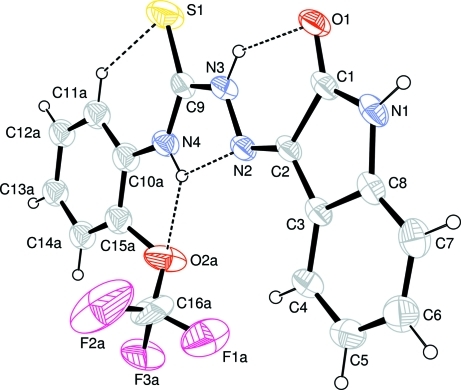
View of the title compound with the atom numbering scheme. The thermal displacements are drawn at the 30% probability level. The dotted lines indicate the intra-molecular H-bondings. Only the majority group of disordered atoms are shown for clarity.

**Fig. 2.**
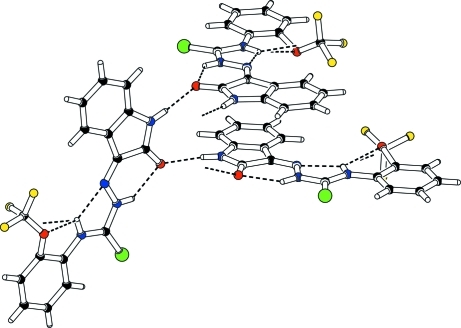
The partial packing (PLATON; Spek, 2009) which shows that molecules form polymeric chains with different ring motifs.

## supplementary crystallographic information

### Comment

In continuation of our work on the synthesis of biologically important isatin derivatives (Pervez *et al.*, 2007, 2008, 2009, 2010), we report herein the structure and preparation of the title compound (I, Fig. 1).

The crystal structure of (II) *i.e.* 4-(2-fluorophenyl)-1-(2-oxoindolin-3-ylidene) thiosemicarbazide has been published (Pervez *et al.*, 2010). The title compound (I) differs from (II) due to the presence of trifluoromethyl instead of fluoro function at position-2 of the phenyl ring substituted at N^4^ of the thiosemicarbazone moiety.

In (I), the 2-oxoindolin A (C1–C8/N1/O1) and thiosemicarbazide B (N2/N3/C9/S1/N4) groups are planar with r. m. s. deviations of 0.0081 Å and 0.0058 Å, respectively. The dihedral angle between A/B is 3.19 (1)°. The disordered phenyl rings C (C10A—C15A) and D (C10B—C15B) of the disordered trifluoromethoxyphenyl substituant are oriented at a dihedral angle of 9.42 (17)° with each other. The dihedral angle between A/C, B/C, A/D and B/D is 3.24 (3), 3.82 (3), 12.59 (14) and 12.59 (14)°, respectively. Due to intramolecular H-bondings (Table 1, Fig. 1), two S(5) and two S(6) (Bernstein *et al.*, 1995) ring motifs are formed. The molecules form polymeric chains (Fig. 2) due to intermolecular H-bonding of N—H···O type. There exist π–π interactions at a distance of 3.2735 (17), 3.563 (2) and [3.664 (4), 3.688 (3)] Å between the centroids of the heterocyclic rings [symmetry code: 1/2 - *x*, 1/2 - *y*, - *z*], the heterocyclic and majority phenyl ring containing trifluoromethoxy [symmetry code: - *x*, - *y*, - *z*] and phenyl rings containing trifluoromethoxy [symmetry code: 1/2 - *x*, - 1/2 - *y*, - *z*], respectively. The trifluoromethoxyphenyl is disordered over two set of sites with occupancy ratio 0.642 (10):0.358 (10).

### Experimental

To a hot solution of isatin (0.74 g, 5.0 mmol) in ethanol (10 ml) containing a few drops of glacial acetic acid was added 4-*o*-trifluoromethoxyphenylthiosemicarbazide (1.26 g, 5.0 mmol) dissolved in ethanol (10 ml) under stirring. The reaction mixture was then heated under reflux for 2 h. The yellow crystalline solid formed during heating was collected by suction filtration. Thorough washing with hot ethanol followed by ether provided the title compound (I) in pure form (1.51 g, 80%), m.p. 517 K (*d*). The single crystals of (I) were grown in ethyl acetate by slow evaporation at room temperature.

### Refinement

The trifluoromethoxyphenyl is highly disordered. The present refinement is the best one with acceptable bond lengths and refinement parameters. However, the thermal ellipsoids of O2A, F2A and F2B cannot be reduced. The disordered phenyl rings of trifluoromethoxyphenyl are treated as regular hexagones with equal anisotropic thermal parameters. The thermal parameters of disordered C-atoms of trifluoromethoxy are also treated to be equal.

The H-atoms were positioned geometrically (N—H = 0.86, C–H = 0.93 Å) and refined as riding with *U*_iso_(H) = x*U*_eq_(C, N), where x = 1.2 for all H-atoms.

### Crystal data

**Table:**

C_16_H_11_F_3_N_4_O_2_S	*D*_x_ = 1.537 Mg m^−^^3^
*M**_r_* = 380.35	Mo *K*α radiation, λ = 0.71073 Å
Tetragonal, *P*4_2_/*n*	Cell parameters from 2321 reflections
Hall symbol: -P 4bc	θ = 3.1–28.3°
*a* = 13.4746 (8) Å	µ = 0.25 mm^−^^1^
*c* = 18.1073 (10) Å	*T* = 296 K
*V* = 3287.7 (3) Å^3^	Prism, yellow
*Z* = 8	0.32 × 0.24 × 0.22 mm
*F*(000) = 1552	

### Data collection

**Table:**

Bruker Kappa APEXII CCD diffractometer	4051 independent reflections
Radiation source: fine-focus sealed tube	2321 reflections with *I* > 2σ(*I*)
graphite	*R*_int_ = 0.030
Detector resolution: 7.50 pixels mm^-1^	θ_max_ = 28.3°, θ_min_ = 3.1°
ω scans	*h* = −17→16
Absorption correction: multi-scan (*SADABS*; Bruker, 2005)	*k* = −16→13
*T*_min_ = 0.937, *T*_max_ = 0.951	*l* = −24→18
15673 measured reflections	

### Refinement

**Table:**

Refinement on *F*^2^	Primary atom site location: structure-invariant direct methods
Least-squares matrix: full	Secondary atom site location: difference Fourier map
*R*[*F*^2^ > 2σ(*F*^2^)] = 0.069	Hydrogen site location: inferred from neighbouring sites
*wR*(*F*^2^) = 0.202	H-atom parameters constrained
*S* = 1.04	*w* = 1/[σ^2^(*F*_o_^2^) + (0.0768*P*)^2^ + 2.277*P*] where *P* = (*F*_o_^2^ + 2*F*_c_^2^)/3
4051 reflections	(Δ/σ)_max_ < 0.001
242 parameters	Δρ_max_ = 0.62 e Å^−^^3^
46 restraints	Δρ_min_ = −0.39 e Å^−^^3^

### Special details

**Table:**

Geometry. All e.s.d.'s (except the e.s.d. in the dihedral angle between two l.s. planes) are estimated using the full covariance matrix. The cell e.s.d.'s are taken into account individually in the estimation of e.s.d.'s in distances, angles and torsion angles; correlations between e.s.d.'s in cell parameters are only used when they are defined by crystal symmetry. An approximate (isotropic) treatment of cell e.s.d.'s is used for estimating e.s.d.'s involving l.s. planes.
Refinement. Refinement of *F*^2^ against ALL reflections. The weighted *R*-factor *wR* and goodness of fit *S* are based on *F*^2^, conventional *R*-factors *R* are based on *F*, with *F* set to zero for negative *F*^2^. The threshold expression of *F*^2^ > σ(*F*^2^) is used only for calculating *R*-factors(gt) *etc*. and is not relevant to the choice of reflections for refinement. *R*-factors based on *F*^2^ are statistically about twice as large as those based on *F*, and *R*- factors based on ALL data will be even larger.

### Fractional atomic coordinates and isotropic or equivalent isotropic displacement parameters (Å^2^)

**Table:**

	*x*	*y*	*z*	*U*_iso_*/*U*_eq_	Occ. (<1)
S1	0.12312 (12)	−0.17880 (9)	−0.07998 (6)	0.1023 (5)	
O1	0.13028 (17)	0.12230 (19)	−0.19341 (11)	0.0617 (6)	
O2A	0.1645 (8)	−0.0082 (5)	0.1781 (4)	0.098 (3)	0.642 (10)
C16A	0.1209 (8)	0.0557 (10)	0.2175 (6)	0.110 (3)	0.642 (10)
F1A	0.1590 (10)	0.1485 (9)	0.2063 (6)	0.142 (4)	0.642 (10)
F2A	0.0226 (6)	0.0520 (8)	0.2108 (9)	0.256 (8)	0.642 (10)
F3A	0.1494 (6)	0.0461 (6)	0.2903 (2)	0.143 (3)	0.642 (10)
O2B	0.1302 (18)	0.0168 (10)	0.1743 (8)	0.146 (9)	0.358 (10)
C16B	0.1390 (16)	0.0527 (18)	0.2389 (9)	0.110 (3)	0.358 (10)
F1B	0.1175 (18)	0.1457 (16)	0.2154 (13)	0.155 (9)	0.358 (10)
F2B	0.230 (2)	0.0142 (14)	0.246 (2)	0.42 (2)	0.358 (10)
F3B	0.0538 (17)	0.0320 (11)	0.2705 (9)	0.184 (9)	0.358 (10)
C1	0.1288 (2)	0.1907 (3)	−0.14785 (15)	0.0506 (7)	
C2	0.1252 (2)	0.1801 (2)	−0.06543 (13)	0.0452 (7)	
C3	0.1269 (2)	0.2802 (2)	−0.03581 (15)	0.0471 (7)	
C4	0.1262 (2)	0.3187 (3)	0.03511 (17)	0.0596 (8)	
H4	0.1239	0.2771	0.0761	0.071*	
C5	0.1289 (3)	0.4203 (3)	0.0433 (2)	0.0715 (10)	
H5	0.1288	0.4474	0.0905	0.086*	
C6	0.1319 (3)	0.4826 (3)	−0.0170 (2)	0.0729 (10)	
H6	0.1332	0.5509	−0.0095	0.087*	
C7	0.1330 (3)	0.4460 (3)	−0.0883 (2)	0.0639 (9)	
H7	0.1351	0.4881	−0.1290	0.077*	
C8	0.1307 (2)	0.3447 (3)	−0.09654 (16)	0.0516 (7)	
C9	0.1229 (2)	−0.0776 (2)	−0.02940 (17)	0.0537 (7)	
C10A	0.1224 (4)	−0.1399 (3)	0.0985 (2)	0.0673 (11)	0.642 (10)
C11A	0.1162 (5)	−0.2412 (3)	0.0844 (2)	0.0673 (11)	0.642 (10)
H11A	0.1136	−0.2640	0.0360	0.081*	0.642 (10)
C12A	0.1139 (4)	−0.3082 (3)	0.1428 (3)	0.0673 (11)	0.642 (10)
H12A	0.1098	−0.3759	0.1333	0.081*	0.642 (10)
C13A	0.1178 (3)	−0.2740 (4)	0.2151 (2)	0.0673 (11)	0.642 (10)
H13A	0.1163	−0.3189	0.2541	0.081*	0.642 (10)
C14A	0.1240 (4)	−0.1728 (4)	0.22919 (19)	0.0673 (11)	0.642 (10)
H14A	0.1266	−0.1500	0.2776	0.081*	0.642 (10)
C15A	0.1263 (4)	−0.1058 (3)	0.1709 (3)	0.0673 (11)	0.642 (10)
C10B	0.1284 (4)	−0.1331 (4)	0.1078 (3)	0.0557 (16)	0.358 (10)
C11B	0.1090 (6)	−0.2343 (4)	0.1039 (4)	0.0557 (16)	0.358 (10)
H11B	0.0957	−0.2637	0.0585	0.067*	0.358 (10)
C12B	0.1096 (6)	−0.2914 (5)	0.1678 (5)	0.0557 (16)	0.358 (10)
H12B	0.0967	−0.3591	0.1652	0.067*	0.358 (10)
C13B	0.1296 (5)	−0.2474 (6)	0.2356 (4)	0.0557 (16)	0.358 (10)
H13B	0.1300	−0.2856	0.2784	0.067*	0.358 (10)
C14B	0.1490 (6)	−0.1462 (6)	0.2395 (3)	0.0557 (16)	0.358 (10)
H14B	0.1623	−0.1167	0.2849	0.067*	0.358 (10)
C15B	0.1484 (5)	−0.0890 (5)	0.1756 (3)	0.0557 (16)	0.358 (10)
N1	0.13202 (18)	0.2883 (2)	−0.16207 (12)	0.0553 (7)	
H1	0.1345	0.3134	−0.2057	0.066*	
N2	0.12204 (17)	0.09849 (19)	−0.02780 (12)	0.0475 (6)	
N3	0.12066 (19)	0.0127 (2)	−0.06555 (13)	0.0528 (7)	
H3	0.1184	0.0140	−0.1130	0.063*	
N4	0.12473 (19)	−0.0670 (2)	0.04415 (13)	0.0553 (7)	
H4A	0.1278	−0.0069	0.0599	0.066*	

### Atomic displacement parameters (Å^2^)

**Table:**

	*U*^11^	*U*^22^	*U*^33^	*U*^12^	*U*^13^	*U*^23^
S1	0.1630 (13)	0.0717 (7)	0.0722 (7)	−0.0081 (7)	0.0368 (7)	−0.0177 (5)
O1	0.0676 (15)	0.0853 (16)	0.0323 (10)	−0.0031 (12)	0.0074 (9)	−0.0023 (10)
O2A	0.190 (8)	0.054 (4)	0.051 (3)	0.024 (4)	0.019 (4)	0.005 (3)
C16A	0.151 (8)	0.120 (6)	0.060 (6)	−0.030 (5)	−0.059 (5)	−0.016 (5)
F1A	0.180 (11)	0.123 (6)	0.123 (7)	−0.011 (6)	−0.038 (7)	−0.019 (4)
F2A	0.109 (5)	0.203 (10)	0.457 (19)	0.043 (5)	−0.092 (9)	−0.163 (12)
F3A	0.179 (7)	0.215 (7)	0.034 (2)	−0.028 (6)	−0.003 (3)	−0.027 (3)
O2B	0.33 (2)	0.069 (7)	0.044 (5)	0.121 (10)	0.026 (8)	0.004 (4)
C16B	0.151 (8)	0.120 (6)	0.060 (6)	−0.030 (5)	−0.059 (5)	−0.016 (5)
F1B	0.19 (2)	0.112 (10)	0.159 (12)	−0.054 (10)	0.060 (12)	−0.080 (9)
F2B	0.42 (4)	0.170 (16)	0.69 (5)	−0.075 (19)	−0.42 (4)	0.02 (2)
F3B	0.25 (2)	0.151 (10)	0.152 (13)	0.005 (14)	0.136 (15)	−0.024 (10)
C1	0.0423 (16)	0.078 (2)	0.0314 (13)	0.0001 (14)	0.0041 (11)	0.0070 (14)
C2	0.0400 (15)	0.0660 (19)	0.0296 (12)	−0.0008 (13)	0.0025 (10)	0.0047 (12)
C3	0.0425 (15)	0.0615 (18)	0.0374 (14)	0.0019 (13)	0.0042 (11)	0.0062 (12)
C4	0.068 (2)	0.069 (2)	0.0415 (16)	0.0017 (17)	0.0063 (14)	0.0006 (14)
C5	0.085 (3)	0.071 (2)	0.059 (2)	0.0015 (19)	0.0102 (18)	−0.0106 (17)
C6	0.073 (2)	0.064 (2)	0.082 (3)	0.0055 (18)	0.0098 (19)	−0.0010 (19)
C7	0.059 (2)	0.063 (2)	0.070 (2)	0.0048 (16)	0.0044 (16)	0.0183 (17)
C8	0.0412 (16)	0.071 (2)	0.0429 (15)	0.0018 (14)	0.0043 (12)	0.0121 (13)
C9	0.0481 (17)	0.064 (2)	0.0486 (16)	−0.0024 (14)	0.0112 (13)	−0.0015 (14)
C10A	0.0693 (19)	0.073 (2)	0.0594 (17)	−0.0060 (14)	0.0037 (13)	0.0070 (12)
C11A	0.0693 (19)	0.073 (2)	0.0594 (17)	−0.0060 (14)	0.0037 (13)	0.0070 (12)
C12A	0.0693 (19)	0.073 (2)	0.0594 (17)	−0.0060 (14)	0.0037 (13)	0.0070 (12)
C13A	0.0693 (19)	0.073 (2)	0.0594 (17)	−0.0060 (14)	0.0037 (13)	0.0070 (12)
C14A	0.0693 (19)	0.073 (2)	0.0594 (17)	−0.0060 (14)	0.0037 (13)	0.0070 (12)
C15A	0.0693 (19)	0.073 (2)	0.0594 (17)	−0.0060 (14)	0.0037 (13)	0.0070 (12)
C10B	0.050 (3)	0.072 (3)	0.045 (2)	0.003 (2)	0.0068 (18)	0.020 (2)
C11B	0.050 (3)	0.072 (3)	0.045 (2)	0.003 (2)	0.0068 (18)	0.020 (2)
C12B	0.050 (3)	0.072 (3)	0.045 (2)	0.003 (2)	0.0068 (18)	0.020 (2)
C13B	0.050 (3)	0.072 (3)	0.045 (2)	0.003 (2)	0.0068 (18)	0.020 (2)
C14B	0.050 (3)	0.072 (3)	0.045 (2)	0.003 (2)	0.0068 (18)	0.020 (2)
C15B	0.050 (3)	0.072 (3)	0.045 (2)	0.003 (2)	0.0068 (18)	0.020 (2)
N1	0.0531 (15)	0.0783 (19)	0.0344 (12)	0.0027 (13)	0.0047 (10)	0.0154 (12)
N2	0.0452 (13)	0.0629 (16)	0.0345 (12)	−0.0010 (11)	0.0049 (9)	0.0009 (11)
N3	0.0606 (16)	0.0635 (17)	0.0342 (12)	−0.0020 (12)	0.0057 (10)	−0.0019 (11)
N4	0.0656 (17)	0.0577 (16)	0.0427 (13)	−0.0005 (13)	0.0059 (11)	0.0042 (11)

### Geometric parameters (Å, °)

**Table:**

S1—C9	1.643 (3)	C9—N3	1.382 (4)
O1—C1	1.237 (4)	C10A—C11A	1.3900
O2A—C16A	1.263 (12)	C10A—C15A	1.3900
O2A—C15A	1.418 (8)	C10A—N4	1.391 (4)
C16A—F2A	1.331 (12)	C11A—C12A	1.3900
C16A—F1A	1.367 (13)	C11A—H11A	0.9300
C16A—F3A	1.380 (9)	C12A—C13A	1.3900
O2B—C16B	1.271 (17)	C12A—H12A	0.9300
O2B—C15B	1.448 (11)	C13A—C14A	1.3900
C16B—F3B	1.314 (19)	C13A—H13A	0.9300
C16B—F2B	1.337 (19)	C14A—C15A	1.3900
C16B—F1B	1.35 (2)	C14A—H14A	0.9300
C1—N1	1.342 (4)	C10B—C11B	1.3900
C1—C2	1.500 (4)	C10B—C15B	1.3900
C2—N2	1.295 (4)	C10B—N4	1.457 (4)
C2—C3	1.452 (4)	C11B—C12B	1.3900
C3—C4	1.385 (4)	C11B—H11B	0.9300
C3—C8	1.403 (4)	C12B—C13B	1.3900
C4—C5	1.377 (5)	C12B—H12B	0.9300
C4—H4	0.9300	C13B—C14B	1.3900
C5—C6	1.378 (5)	C13B—H13B	0.9300
C5—H5	0.9300	C14B—C15B	1.3900
C6—C7	1.383 (5)	C14B—H14B	0.9300
C6—H6	0.9300	N1—H1	0.8600
C7—C8	1.373 (5)	N2—N3	1.342 (3)
C7—H7	0.9300	N3—H3	0.8600
C8—N1	1.409 (4)	N4—H4A	0.8600
C9—N4	1.340 (4)		
			
C16A—O2A—C15A	121.0 (9)	C10A—C11A—H11A	120.0
O2A—C16A—F2A	112.7 (9)	C12A—C11A—H11A	120.0
O2A—C16A—F1A	111.4 (11)	C13A—C12A—C11A	120.0
F2A—C16A—F1A	113.3 (11)	C13A—C12A—H12A	120.0
O2A—C16A—F3A	110.2 (10)	C11A—C12A—H12A	120.0
F2A—C16A—F3A	111.1 (10)	C12A—C13A—C14A	120.0
F1A—C16A—F3A	97.1 (9)	C12A—C13A—H13A	120.0
C16B—O2B—C15B	110.1 (16)	C14A—C13A—H13A	120.0
O2B—C16B—F3B	103.8 (18)	C13A—C14A—C15A	120.0
O2B—C16B—F2B	91.6 (18)	C13A—C14A—H14A	120.0
F3B—C16B—F2B	133 (3)	C15A—C14A—H14A	120.0
O2B—C16B—F1B	92.5 (15)	C14A—C15A—C10A	120.0
F3B—C16B—F1B	98 (2)	C14A—C15A—O2A	122.7 (4)
F2B—C16B—F1B	126 (2)	C10A—C15A—O2A	114.1 (5)
O1—C1—N1	127.0 (3)	C11B—C10B—C15B	120.0
O1—C1—C2	126.4 (3)	C11B—C10B—N4	123.6 (3)
N1—C1—C2	106.5 (3)	C15B—C10B—N4	116.4 (3)
N2—C2—C3	126.5 (2)	C12B—C11B—C10B	120.0
N2—C2—C1	127.2 (3)	C12B—C11B—H11B	120.0
C3—C2—C1	106.2 (2)	C10B—C11B—H11B	120.0
C4—C3—C8	119.7 (3)	C13B—C12B—C11B	120.0
C4—C3—C2	133.7 (3)	C13B—C12B—H12B	120.0
C8—C3—C2	106.6 (2)	C11B—C12B—H12B	120.0
C5—C4—C3	118.2 (3)	C12B—C13B—C14B	120.0
C5—C4—H4	120.9	C12B—C13B—H13B	120.0
C3—C4—H4	120.9	C14B—C13B—H13B	120.0
C4—C5—C6	121.4 (3)	C13B—C14B—C15B	120.0
C4—C5—H5	119.3	C13B—C14B—H14B	120.0
C6—C5—H5	119.3	C15B—C14B—H14B	120.0
C5—C6—C7	121.6 (4)	C14B—C15B—C10B	120.0
C5—C6—H6	119.2	C14B—C15B—O2B	124.1 (6)
C7—C6—H6	119.2	C10B—C15B—O2B	111.9 (7)
C8—C7—C6	117.1 (3)	C1—N1—C8	111.5 (2)
C8—C7—H7	121.5	C1—N1—H1	124.2
C6—C7—H7	121.5	C8—N1—H1	124.2
C7—C8—C3	122.1 (3)	C2—N2—N3	117.6 (2)
C7—C8—N1	128.8 (3)	N2—N3—C9	121.1 (2)
C3—C8—N1	109.1 (3)	N2—N3—H3	119.5
N4—C9—N3	112.2 (3)	C9—N3—H3	119.5
N4—C9—S1	130.0 (3)	C9—N4—C10A	128.9 (3)
N3—C9—S1	117.8 (2)	C9—N4—C10B	136.2 (4)
C11A—C10A—C15A	120.0	C10A—N4—C10B	8.0 (4)
C11A—C10A—N4	124.4 (3)	C9—N4—H4A	115.6
C15A—C10A—N4	115.6 (3)	C10A—N4—H4A	115.6
C10A—C11A—C12A	120.0	C10B—N4—H4A	108.1
			
C15A—O2A—C16A—F2A	−40.2 (14)	C16A—O2A—C15A—C14A	−67.6 (12)
C15A—O2A—C16A—F1A	−168.9 (7)	C16A—O2A—C15A—C10A	132.6 (8)
C15A—O2A—C16A—F3A	84.6 (12)	C15B—C10B—C11B—C12B	0.0
C15B—O2B—C16B—F3B	−80 (2)	N4—C10B—C11B—C12B	−177.1 (2)
C15B—O2B—C16B—F2B	54 (2)	C10B—C11B—C12B—C13B	0.0
C15B—O2B—C16B—F1B	−179.8 (18)	C11B—C12B—C13B—C14B	0.0
O1—C1—C2—N2	−0.7 (5)	C12B—C13B—C14B—C15B	0.0
N1—C1—C2—N2	−179.6 (3)	C13B—C14B—C15B—C10B	0.0
O1—C1—C2—C3	178.8 (3)	C13B—C14B—C15B—O2B	155.7 (14)
N1—C1—C2—C3	−0.1 (3)	C11B—C10B—C15B—C14B	0.0
N2—C2—C3—C4	0.4 (5)	N4—C10B—C15B—C14B	177.3 (2)
C1—C2—C3—C4	−179.2 (3)	C11B—C10B—C15B—O2B	−158.5 (12)
N2—C2—C3—C8	179.8 (3)	N4—C10B—C15B—O2B	18.8 (12)
C1—C2—C3—C8	0.2 (3)	C16B—O2B—C15B—C14B	16 (3)
C8—C3—C4—C5	0.3 (5)	C16B—O2B—C15B—C10B	173.1 (16)
C2—C3—C4—C5	179.6 (3)	O1—C1—N1—C8	−179.0 (3)
C3—C4—C5—C6	0.3 (5)	C2—C1—N1—C8	−0.1 (3)
C4—C5—C6—C7	−0.5 (6)	C7—C8—N1—C1	−179.9 (3)
C5—C6—C7—C8	0.2 (5)	C3—C8—N1—C1	0.3 (3)
C6—C7—C8—C3	0.4 (5)	C3—C2—N2—N3	179.8 (3)
C6—C7—C8—N1	−179.4 (3)	C1—C2—N2—N3	−0.8 (4)
C4—C3—C8—C7	−0.6 (4)	C2—N2—N3—C9	176.3 (3)
C2—C3—C8—C7	179.9 (3)	N4—C9—N3—N2	1.1 (4)
C4—C3—C8—N1	179.2 (3)	S1—C9—N3—N2	−178.9 (2)
C2—C3—C8—N1	−0.3 (3)	N3—C9—N4—C10A	176.6 (3)
C15A—C10A—C11A—C12A	0.0	S1—C9—N4—C10A	−3.5 (5)
N4—C10A—C11A—C12A	−179.7 (4)	N3—C9—N4—C10B	−179.2 (4)
C10A—C11A—C12A—C13A	0.0	S1—C9—N4—C10B	0.7 (6)
C11A—C12A—C13A—C14A	0.0	C11A—C10A—N4—C9	−0.9 (5)
C12A—C13A—C14A—C15A	0.0	C15A—C10A—N4—C9	179.4 (3)
C13A—C14A—C15A—C10A	0.0	C11A—C10A—N4—C10B	−159 (3)
C13A—C14A—C15A—O2A	−158.6 (7)	C15A—C10A—N4—C10B	21 (2)
C11A—C10A—C15A—C14A	0.0	C11B—C10B—N4—C9	−14.8 (5)
N4—C10A—C15A—C14A	179.8 (4)	C15B—C10B—N4—C9	168.0 (4)
C11A—C10A—C15A—O2A	160.3 (6)	C11B—C10B—N4—C10A	10 (2)
N4—C10A—C15A—O2A	−19.9 (6)	C15B—C10B—N4—C10A	−168 (3)

### Hydrogen-bond geometry (Å, °)

**Table:**

*D*—H···*A*	*D*—H	H···*A*	*D*···*A*	*D*—H···*A*
N1—H1···O1^i^	0.86	1.99	2.841 (3)	173
N3—H3···O1	0.86	2.07	2.748 (3)	136
N4—H4A···O2A	0.86	2.20	2.607 (8)	109
N4—H4A···N2	0.86	2.13	2.583 (4)	112
C11A—H11A···S1	0.93	2.40	3.096 (4)	132

Symmetry codes: (i) *y*, −*x*+1/2, −*z*−1/2.
